# Comment on ‘Renewed interest in the progesterone receptor in breast cancer’

**DOI:** 10.1038/bjc.2017.90

**Published:** 2017-04-11

**Authors:** Giovanni Simone, Sergio Diotaiuti, Maria Digennaro, Domenico Sambiasi, Simona De Summa, Stefania Tommasi, Rosanna Altieri, Annita Mangia, Caterina Dantona, Angelo Paradiso

**Affiliations:** 1 Pathology Unit, Istituto Tumori Giovanni Paolo II, IRCCS, National Cancer Research Institute, via O Flacco 65, Bari I-70124, Italy; 2 Senology Unit, Istituto Tumori Giovanni Paolo II, IRCCS, National Cancer Research Institute, via O Flacco 65, Bari I-70124, Italy; 3 Experimental Medical Oncology, Istituto Tumori Giovanni Paolo II, IRCCS, National Cancer Research Institute, via O Flacco 65, Bari I-70124, Italy; 4 Molecular Genetics, Istituto Tumori Giovanni Paolo II, IRCCS, National Cancer Research Institute, via O Flacco 65, Bari I-70124, Italy; 5 Functional Biomorphology, Istituto Tumori Giovanni Paolo II, IRCCS, National Cancer Research Institute, via O Flacco 65, Bari I-70124, Italy; 6 Department of General Surgery,Ospedale Civico di Lugano, Via Tesserete 46, 6900 Lugano, Switzerland

**Sir**,

The present letter refers to the Editorial by Lim *et al*, on clinical relevance of progesterone receptor (PgR) expression in breast cancer, which was recently published in British Journal of Cancer (Lim *et al*, 2016). The Editorial by Lim *et al* stated the need to reconsider the value of PgR as a prognostic-predictive factor in both the adjuvant and metastatic settings that has ‘to date not been demonstrated’ (Lim *et al*, 2016). In effect, the debate around the clinical value of PgR has been renewed by recent results describing its prognostic relevance when included in an endocrine receptor score (Campbell *et al*, 2016), the substantial crosstalk between the estrogen receptor (ER) and PgR signaling pathways (Mohammed *et al*, 2015) and new progestins in experimental phase (Esber *et al*, 2016).

It remains out from this debate one of the most modern aspects of the standard treatment for primary breast cancer, that is, the management of sentinel lymph node biopsy (SLNB) in patients with early disease. In fact, nowadays, SLNB is considered the standard approach for axillary staging in patients with clinically non-palpable axillary lymph nodes (Lyman *et al*, 2016). However, the need for availability of powerful predictive markers of disease involvement of locoregional nodes is still stressed by the fact that, in patients with clinically negative axilla, the successive demonstration of axillary node involvement by SLNB is generally low, thus ensuring that this technique is not strictly needed for a large proportion of cases. In recent retrospective studies analysing small series of patients, the only factors predicting SLNB metastatisation were age and body mass index at disease diagnosis (Thangarajah *et al*, 2016) and Ki-67 tumour expression (Ozemir *et al*, 2016).

In order to look for clinical–pathological tumour characteristics predicting the pathological status of the axilla, we retrospectively analysed breast cancer cases consecutively treated with primary surgery between 2008–2014 at our National Cancer Research Centre of Bari. Data from 2002 patients were collected; 1297 (64.78%) cases resulted characterised by clinically positive axillary nodes and then received complete axillary lymph node dissection. The remaining 705 (35, 22%) cases, with clinically negative axillary nodes, received SLNB and entered the present analysis. ER, PgR, and Mib-1 biomarkers were analysed by immunoistochemical assays and categorised according to standard literature scores already utilised by our team (Paradiso *et al*, 2013). HER2/neu oncogene status was determined according to ASCO/CAP guidelines (Lim *et al*, 2016).

In detail, the group of patients who received SLNB were aged ⩾59 years in 44.2% of cases, T2 in 31.2% of cases, ER positive (>1% of positive tumour cells) in 84% of tumours, and PgR positive (>1% positive tumour cells) in 72.9% of tumours, with high expression of Mib-1 (⩾14% of positive tumour cells) in 48.7% of tumours; finally, 14.2% of tumours resulted in HER2/neu amplified according to IHC/FISH complementary analysis.

One hundred and thirty out of the 705 cases treated with SLNB showed metastasis at least in one of the cleared nodes (median number of cleared nodes=2.3). At univariate analysis, young age (odds ratio (OR) 0.46; 95% confidence interval (CI): 0.3–0.7; *P*=0.003), T2 size (OR: 1.75; 95% CI:1.12–2.7; *P*=0.01) and high Mib1 expression (OR: 1.53, 95% CI: 1.04–2.27, *P*=0.03) resulted significantly associated with the presence of pathological involvement of axillary nodes at SLNB. When a multivariate analysis was performed by a backward stepwise selection using the stepAIC function from the R MASS package (Venables and Ripley, 2002), age at diagnosis, tumour size, PgR status and Mib-1 expression were selected as still statistically significant, with model fitting results shown in Table 1; in specific, PgR-positive status confirmed to be significantly predictive of negative SLNB with an OR of 1.93 (95% CI: 1.12–3.46; *P*=0.02).

Even more interesting, when possible interaction between the variables selected in the first analysis was considered (backward/forward stepwise selection), only PgR-positive status confirmed to be associated independently from other variables with SLNB status (OR: 0.37; 95% CI: 0.17–0.72; *P*<0.005) while a significant interaction became evident between tumour size and Mib-1 expression (Table 1). This last evidence suggests PgR as the only tumour biomarker independently related with SLNB status while higher tumour-proliferative activity and large tumour size have to be considered together to significantly predict locoregional disease spread. In conclusion, in a large, monoinstitutional, and consecutive series of breast cancer patients, we showed that PgR-positive status is a powerful and independent indicator of negative axilla involvement in patients with clinically negative regional nodes and this could be taken into consideration when clinical strategy has to be defined.

## Figures and Tables

**Table 1:** Logistic regression analysis with the dependent variable pathological positive axillary node status at SLNB

**Retained variables**	**OR (95% CI)**	***P*** **-value**
**(a) Logistic regression (backward stepwise procedure)**		
Age ⩾59 years	0.56 (0.34–0.89)	0.016
Tumour size >20 mm	1.68 (1.063–2.66)	0.025
PgR negative^a^	1.93 (1.12–3.46)	0.02
Mib-1 high^a^	1.72 (1.07–2.79)	0.025
**(b) Logistic regression (‘both’ stepwise procedure for interaction analysis)**		
PgR negative	2.69 (1.37–5.67)	0.005
Mib-1 high/tumour size large	2.51 (0.59–3.54)	0.018

Abbreviations: CI, confidence interval; OR, odds ratio; PgR, progesterone receptor; SLNB, sentinel lymph node biopsy. Variables selected by backward stepwise procedure (a) and by ‘both’ stepwise procedures (b) for interaction analysis.

a See text for definition.
